# Enhanced spectral resolution for correlated spectroscopic imaging using inner-product and covariance transform: a pilot analysis of metabolites and lipids in breast cancer in vivo

**DOI:** 10.1038/s41598-023-43356-8

**Published:** 2023-10-05

**Authors:** Ajin Joy, M. Albert Thomas

**Affiliations:** 1 Radiological Sciences, David Geffen School of Medicine at UCLA, 10945 Peter V Ueberroth Building, Suite#1417A, Los Angeles, CA 90095 USA; 2 Physics and Biology in Medicine IDP, University of California Los Angeles, Los Angeles, CA USA; 3 BioEngineering, University of California Los Angeles, Los Angeles, CA USA

**Keywords:** Diagnostic markers, Medical research

## Abstract

Acquisition duration of correlated spectroscopy in vivo can be longer due to a large number of t_1_ increments along the indirect (F_1_) dimension. Limited number of t_1_ increments on the other hand leads to poor spectral resolution along F_1_. Covariance transformation (CT) instead of Fourier transform along t_1_ is an alternative way of increasing the resolution of the 2D COSY spectrum. Prospectively undersampled five-dimensional echo-planar correlated spectroscopic imaging (EP-COSI) data from ten malignant patients and ten healthy women were acquired and reconstructed using compressed sensing. The COSY spectrum at each voxel location was then generated using FFT, CT and a variant of CT called Inner Product (IP). Metabolite and lipid ratios were computed with respect to water from unsuppressed one-dimensional spectrum. The effects of t_1_-ridging artifacts commonly seen with FFT were not observed with CT/IP. Statistically significant differences were observed in the fat cross peaks measured with CT/IP/FFT. Spectral resolution was increased ~ 8.5 times (~ 19.53 Hz in FFT, ~ 2.32 Hz in CT/IP) without affecting the spectral width along F_1_ was possible with CT/IP. CT and IP enabled substantially increased F_1_ resolution effectively with significant gain in scan time and reliable measure of unsaturation index as a biomarker for malignant breast cancer.

## Introduction

MR spectroscopy (MRS) is an efficient biochemical tool for quantifying metabolite and lipid concentrations non-invasively in human breast tissues^1–9^. Altered biochemical concentrations in the malignant breast tissues compared to that of healthy ones have been reported in various breast cancer studies using MRS. In addition to the lipids, water and total choline measures that are commonly reported by one dimensional (1D) in vivo MRS^1–9^, two-dimensional (2D) in vivo MRS has also reported the measures of glycine (Gly), myo-Inositol (mI), saturated and unsaturated lipids and lipid unsaturation as potential biomarkers that can be used to identify malignancy in breast tissues. 2D correlated spectroscopy (COSY) is known to provide better spectral dispersion as compared to 1D, since the J-coupled multiplet resonance peaks are dispersed over two spectral dimensions as opposed to one spectral dimension in 1D MRS. The level of lipid unsaturation as a result, can be measured as the ratio of unsaturated fatty acid cross-peaks arising from J-coupling of olefinic to methylene protons in the 2D COSY spectra.

The 2D spectra were initially recorded from single volume of interest (VOI) using localized correlated spectroscopy (L-COSY) technique^10–12^. It was later extended to MR spectroscopic imaging (MRSI) sequences that record 2D COSY spectra in multiple locations at clinically feasible times, with the help of non-uniform sampling (NUS) and Compressed Sensing (CS) reconstruction^13,14^. Recently, a five-dimensional (5D) echo-planar correlated spectroscopic imaging (EP-COSI) technique combining 2 spectral and 3 spatial dimensions further increased the coverage area by measuring 2D spectra from multiple voxels within multiple slices, during a single scan session, and therefore helps to better localize the malignant tissues across the breast^15,16^.

While the multi-voxel spectroscopic imaging covers a larger area of the breast, the spectral resolution along indirect F_1_ (t_1_) spectral dimension is generally low due to the scan time limitations. Combined with artifacts due to t_1_-ridging caused by factors like subject motion and instrumental fluctuations^17^, this can lead to loss of/corrupted cross peaks, limiting the full potential of the technique. While zero-filling can improve the resolution by interpolation to some extent^18^, it doesn’t improve the separation between different resonant frequencies. It can also introduce ringing artifacts along the F_1_ domain and does not mitigate t_1_-ridging as well.

Recently, covariance NMR^19,20^ has been applied to J-resolved spectroscopic imaging in vivo for increased F_1_ spectral resolution without introducing ringing artifacts^21^. It replaced the second Fourier transformation applied to the t_1_ dimension with a covariance transformation (CT). The resultant spectrum has a spectral resolution in the indirect dimension equal to that in the direct dimension. Therefore, fewer number of t_1_ increments were required to extract the spin correlations than what is required in a conventional FFT based spectral analysis. This facilitates reducing the scan time while achieving higher spectral resolution along F_1_^22^.

Although this approach has been used in various NMR experiments like total correlation spectroscopy (TOCSY) and nuclear Overhauser effect spectroscopy (NOESY)^23–25^, the adaptation to in vivo has been limited. A variant of the covariance NMR spectroscopy called inner-product (IP) NMR spectroscopy, is yet another approach which further improves the covariance NMR by making it robust against changes in the carrier frequency^26^. In this work, we applied the CT and IP approaches to 5D EP-COSI in-vivo to show its advantages over the conventional FFT based COSY spectrum in terms of both improved spectral resolution and minimal influence of t_1_-ridging, while exploring the possibility of further acceleration in scan time. It is shown the biomarkers such as unsaturation index (UI) quantified from the COSY cross peaks may be unambiguously determined from a CT/IP spectrum in the presence of t_1_-ridging.

## Materials and methods

### Subjects

Ten malignant breast masses (n = 10, mean age 52 [range: 41–71] years; *grade-3* (n = 3), *grade-2* (n = 4) and *grade-1* (n = 3)) and healthy (n = 10, mean age 46 (range:29–60) years) volunteers were recruited. The study was performed in accordance with the Declaration of Helsinki and all the subjects gave consent according to the on-site institutional review board guidelines.

### Data acquisition

The 5D EP-COSI data was acquired on a Siemens 3 T Skyra scanner (Siemens Healthineer, Erlangen, Germany) with a dedicated “receive” 24-channel phase-array breast coil and a body “transmit” coil (FOV: 160 × 160 × 120mm^3^, 1.5 mL voxel volume, TR/TE were 1500/35 ms), running on VE11C software platform. 64 t_1_ points sampled were used along F_1_^15^ with a spectral bandwidth (SW) of 1250 Hz and 512 complex t_2_ points, and a SW of 1190 Hz along F_2_. A three-pulse sequence^27^ was employed before the global water suppression. A non-water suppressed scan with one t_1_ point was acquired for eddy current phase correction and coil combination^28^. Two spatial and one spectral dimensions (k_y_,k_z_,t_1_) were non-uniformly sampled with an exponentially-weighted sampling density along t_1_ and gaussian sampling density along the k_y_-k_z_ plane for an acceleration factor of 8. The total scan time was 28 min and 48 s.

### Data reconstruction

The undersampled 5D EP-COSI data was reconstructed using a Group Sparsity (GS)-based compressed sensing (CS) algorithm^15,29^ to estimate the unacquired samples along the k_y_-k_z_-t_1_ dimensions. The dominant lipid peak around 1.3 ppm was zeroed in the Fourier transform of the non-water suppressed signal to obtain a water-dominant time domain signal for the eddy current phase correction and coil combination. The spectral peak volume integrals were computed as described in^16^. The quantified proton resonances along the diagonal (F_1_-F_2_), and off-diagonal are listed in Table 1.

**Table 1:** Metabolites and lipids identified in the 2D COSY spectra of breast tissues.

Diagonal peaks		Cross-peaks	
Peak label	Locations (F_2_, F_1_) ppm	Peak label	Locations (F_2/_F_x_) ppm
Methyl fat (FMETD)	(0.9, 0.9)	CP1	(0.9, 1.3)
Methylene fat (FAT13)	(1.3, 1.3)	CP2	(1.3, 0.9)
Methylene fat (FAT21)	(2.1, 2.1)	CP3	(1.6, 2.3)
Methylene fat (FAT23)	(2.3, 2.3)	CP4	(2.3,1.6)
Methylene fat (FAT29)	(2.9, 2.9)	CP5	(1.3, 2.1)
Choline (Cho)	(3.2, 3.2)	CP6	(2.1,1.3)
Taurine (Tau)	(3.25, 3.25)	Unsaturated fatty acid cross peak, right lower (UFR_lower)	(2.1, 5.4)
myo-Inositol + Glycine (ml + Gly)	(4.1, 4.1)	Unsaturated fatty acid cross peak, left lower (UFL_lower)	(2.9, 5.4)
Methylene glycerol backbone (MGB42)	(4.3, 4.3)	Triglyceryl fat cross peak lower, (TGF_lower)	(4.2, 5.3)
Water (WAT)	(4.7, 4.7)	Unsaturated fatty acid cross peak, right upper (UFR_upper)	(5.4, 2.1)
Olefinic fat (UFD54)	(5.4, 5.4)	Unsaturated fatty acid cross peak, left upper (UFL_upper)	(5.4, 2.9)
		Triglyceryl fat cross peak upper (TGF_upper)	(5.3, 4.2)

### Covariance and inner-product COSY processing

After the GS-CS reconstruction, a hybrid spectral-spatial data matrix $$\mathbf{D}$$(x, y, z, t_2_, t_1_ $$)$$ was outputted where x, y and z were the Fourier transform of k_x_, k_y_ and k_z_ dimensions in k-space. After Fourier transforming the direct spectral dimension (t_2_), we get the mixed time–frequency matrix $$\mathbf{A}$$(F_2_, t_1_) for every special location (x = 1, 2, 3…, 16; y = 1, 2, 3…, 16; z = 1, 2, 3…, 8), with a stack of 1D spectra. Fourier transforming the indirect dimension t_1_ of $$\mathbf{A}$$ yielded the conventional Fourier transformed COSY spectrum $$\mathbf{S}$$(F_2_, F_1_ ) of size (512 × 64).

A covariance transform was instead obtained from **A** in the following manner^25^.

**Step 1** Make matrix $\mathbf{A}$ offset free by subtracting the average 1D spectrum (${\mathbf{A}}_{\mathrm{avg}}$) from it.

$\widetilde{\mathbf{A}} = \mathbf{A}-{\mathbf{A}}_{\mathrm{avg}}$. This 1D spectrum is formed by averaging over the t_1_ dimension in $\mathbf{A}$.

**Step 2** Apply a singular value decomposition (SVD)^30^ to the transposed mixed time–frequency matrix ${\widetilde{\mathbf{A}}}^{\mathrm{T}}$= $\widetilde{\mathbf{U}}$.$\widetilde{\mathbf{W}}$.${\widetilde{\mathbf{V}}}^{\mathrm{T}}$, where $\widetilde{\mathbf{U}}$ and $\widetilde{\mathbf{V}}$ are the singular vectors and $\widetilde{\mathbf{W}}$ is the diagonal matrix with singular values as its diagonal elements.

**Step 3** The final covariance transformed spectrum is then calculated as, ${\mathbf{S}}_{{{\mathbf{cov}}}} = \left( {{\tilde{\mathbf{A}}}^{{\text{T}}} .{\tilde{\mathbf{A}}}} \right)^{1/2} = { }{\tilde{\mathbf{U}}}.{\tilde{\mathbf{W}}}.{\tilde{\mathbf{V}}}^{{\text{T}}}$, which gives a high-resolution spectrum of size (512 × 512), with the spectral width along indirect spectral dimension same as that of the direct spectral dimension.

The similarity between covariance and Fourier-transformed spectra is based on the Parseval’s theorem as described in^20^. It follows that the covariance spectrum ${\mathbf{C}} = {\tilde{\mathbf{A}}}^{{\text{T}}} .{\mathbf{A}}$ correspond to the 2D spectrum squared, i.e., ${\mathbf{S}}^{2}$, provided that the ${\mathbf{A}}_{{{\text{avg}}}}$ in the calculation of covariance vanishes. However, it is shown later that the condition of vanishing average might not hold near the spectral center due to the relatively slow spin precision, as well as when reducing the number of t_1_ points^26^. It is shown that this problem can be overcome by discarding the average. i.e., by choosing ${\tilde{\mathbf{A}}} = {\mathbf{A}}$ in step 1. This is called the inner-product covariance transform where the Parseval’s theorem assures the correspondence between a matrix ${\mathbf{I}} = {\mathbf{A}}^{{\text{T}}} .{\mathbf{A}}$ and ${\mathbf{S}}^{2}$, even if the average doesn’t vanish^26^. High resolution IP covariance matrix is therefore calculated as ${\mathbf{S}}_{{{\mathbf{IP}}}} { } = { }\left( {{\mathbf{A}}^{{\text{T}}} .{\mathbf{A}}} \right)^{1/2} { } = { }{\mathbf{U}}.{\mathbf{W}}.{\mathbf{U}}^{{\text{T}}}$, where ${\mathbf{U}}$, ${\mathbf{V}}$ and ${\mathbf{W}}$ are the singular vectors singular values of ${\mathbf{A}}^{{\text{T}}}$.

Even though high resolution can be achieved by CT while also minimizing t_1_-ridging, reduced number of t_1_ points can introduce spurious correlations in the CT/IP spectrum. However, the deterministic nature of the sampling scheme and resonant positions enable us to mask out these spurious correlations while retaining the advantage in resolution^31^. This may be approached in multiple ways. One option is to determine these spurious correlations based on their intensity, frequencies, and t_1_ sampling as shown in^31^. Another option is to use a map of true resonance frequencies obtained via high resolution FFT as a reference to identify spurious correlations. In this work, we used the resonance frequency locations identified from a prior knowledge based synthetic spectra to remove the spurious correlations.

### Statistical analysis

Means, standard deviations, and 95% confidence intervals were calculated for each metabolite and lipid in IP, CT and FFT spectra. Analysis of variance procedures including Brown-Forsythe was used to test for the equality of means between the quantified IP, CT and FFT. Games-Howell and Tukey HSD multiple comparisons were used to determine the pair-wise significance among the three different groups based on the homogeneity of variances. Student’s t-test was used to compare the means of metabolite and lipid ratios between malignant and healthy tissues.

### Ethical approval and informed consent

The study was approved by the institutional review board of the University of California, Los Angeles. The subjects in the study provided written informed consent.

## Results

### FFT versus CT versus IP

**Figure 1:**
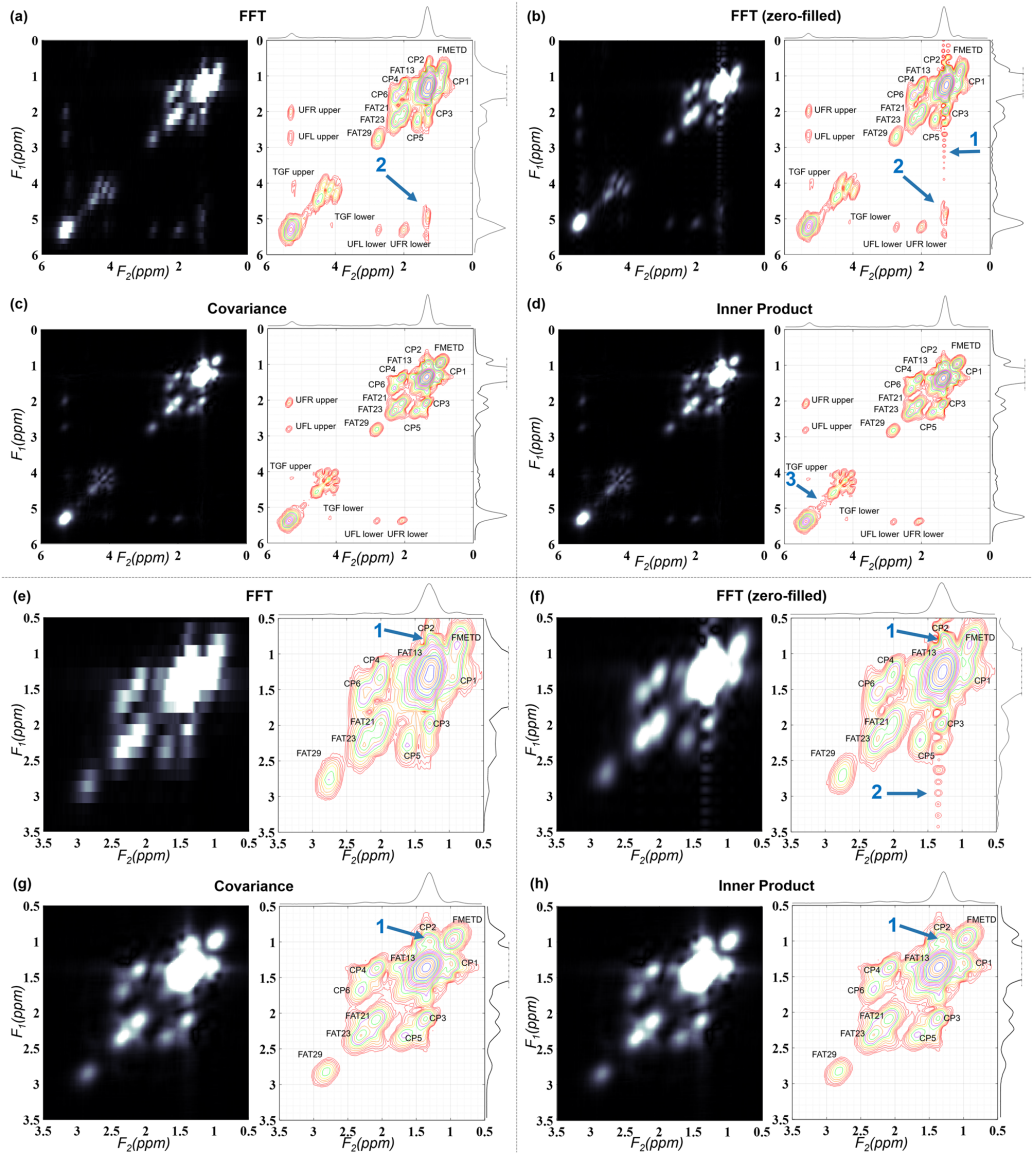
Reconstructed 2D COSY spectrum of healthy tissues from a 60-year-old woman. (**a**) Intensity and contour plots of FFT based spectra (0–6 ppm). Arrow 2 indicates false peak from t_1_-ridging. (**b**) Intensity and contour plots (0–6 ppm) of FFT based spectra after zero-filling t_1_ dimension to 512 points. Arrow 1 shows the ringing effect. (**c**) Intensity and contour plots of CT based spectra (0–6 ppm). (**d**) Intensity and contour plots of IP based spectra (0–6 ppm). Arrow 3 points out the region where IP spectrum is different from CT spectrum. (**e**) Intensity and contour plots of FFT based spectra (0.5–3.5 ppm). Arrow 1 indicates altered lipid cross-peak from t_1_-ridging. (**f**) Intensity and contour plots (0.5–3.5 ppm) of FFT based spectra after zero-filling along the t_1_ dimension to 512 points. Arrow 2 shows the ringing effect. (**g**) Intensity and contour plots of CT based spectra (0.5–3.5 ppm). (**h**) Intensity and contour plots of the IP based spectra (0.5–3.5 ppm).

A 2D spectrum from a healthy subject generated using FFT, FFT with zero-filling along t_1_, covariance transform and inner product are compared in Fig. 1 as intensity plots and contour plots. FFT based spectrum shown in Fig. 1a without zero-filling had a matrix size of 512 × 64 while zero-filled FFT, CT and IP in Fig. 1b–d had matrix size of 512 × 512. Only 0–6 ppm range is shown in the figure. While FFT and zero-filled FFT had a SW of 1250 Hz along F_1_, both CT and IP had a SW of 1190 Hz. All four spectra had a SW of 1190 Hz along F_2_. Arrow 1 indicates the ringing caused by zero-padding in zero-filled FFT. Arrow 2 is pointed at the false peaks in FFT and zero-filled FFT which is in fact due to a mild t_1_-ridging. Arrow 3 shows the region where IP based spectra differed from the CT.

Panels (e–h) show enlarged regions from figures in panels (a–d) comprising 0.5–3.5 ppm along both axes. Apart from the ringing indicated by arrow 1 in zero-filled FFT, this figure shows the effect of t_1_-ridging on the cross-peaks between 0.9 and 1.3 ppm, as pointed out by arrow 1 in (e, f) and the lack of this degradation in (g, h).

**Figure 2:**
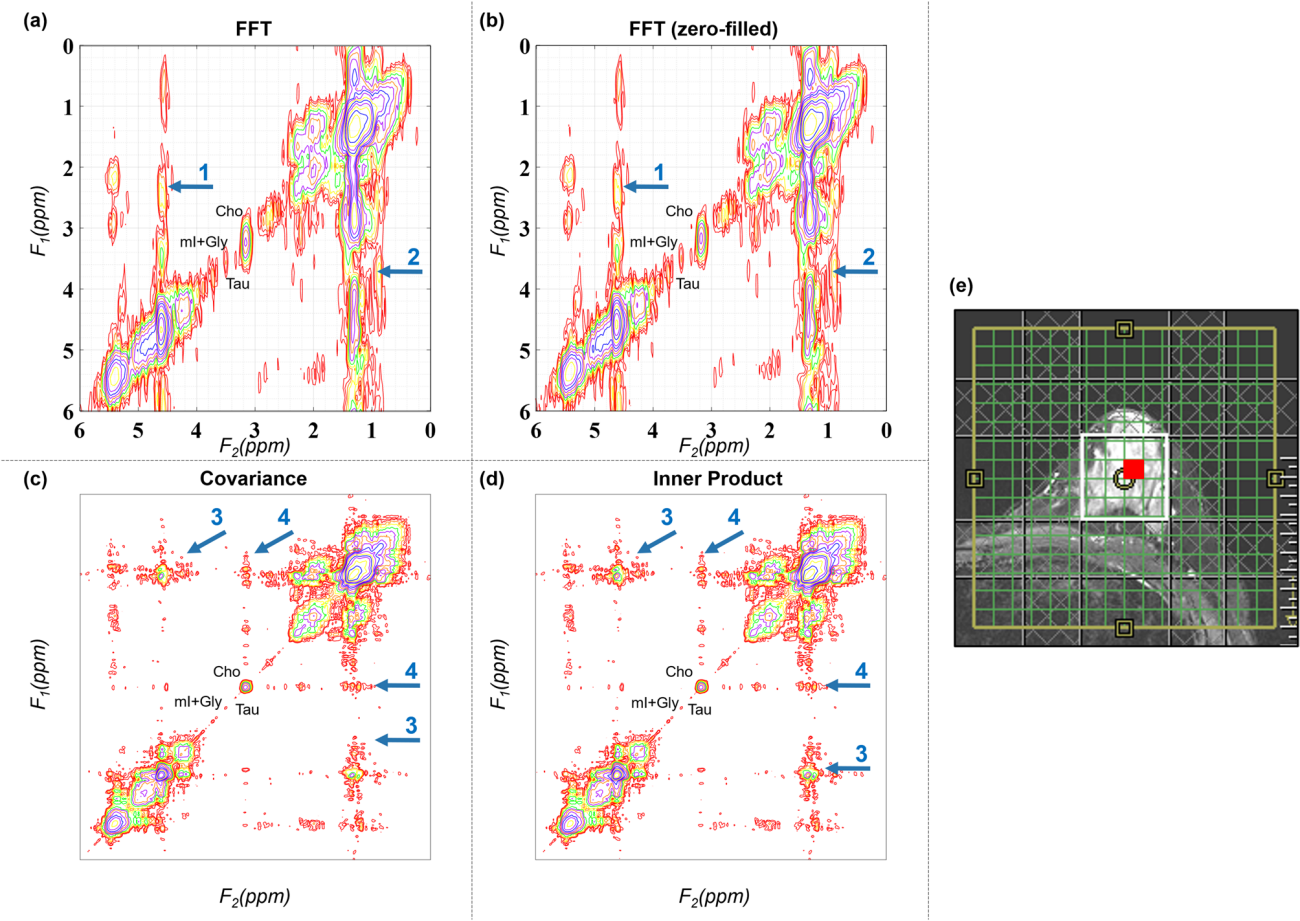
Reconstructed 2D COSY spectra from a malignant lesion identified in 45-year-old patient (grade 3 invasive ductal carcinoma, estrogen receptor positive, progesterone receptor positive, her2 positive, ki-67 = 20% and BI-RADS 5): (**a**) FFT based spectrum (**b**) FFT based spectrum after zero-filling along t_1_, (**c**) CT based spectrum, (**d**) IP based spectrum, (**e**) T_2_-weighted MR image with the white box representing the VOI placement. Red square shows the location of extracted spectrum. Arrows 1 and 2 point out the effect of t_1_-ridging in (**a**) and (**b**). Examples of spurious correlation appearing in CT and IP are pointed out by arrows 3 and 4.

Another 2D spectrum from a malignant lesion identified in a 45-year-old patient is shown in Fig. 2. Sections (a)–(d) shows the 2D spectra generated using FFT, FFT with zero-filling along t_1_, CT and IP. T_2_-weighted MR image with the white box representing the VOI placement is shown in (e). Red square shows the location of the extracted 2D spectrum. Arrows 1 and 2 points out the stronger effect of t_1_-ridging in (a) and (b) as compared to Fig. 1. Examples of spurious correlation appearing in CT and IP are pointed out by arrows 3 and 4.

**Figure 3:**
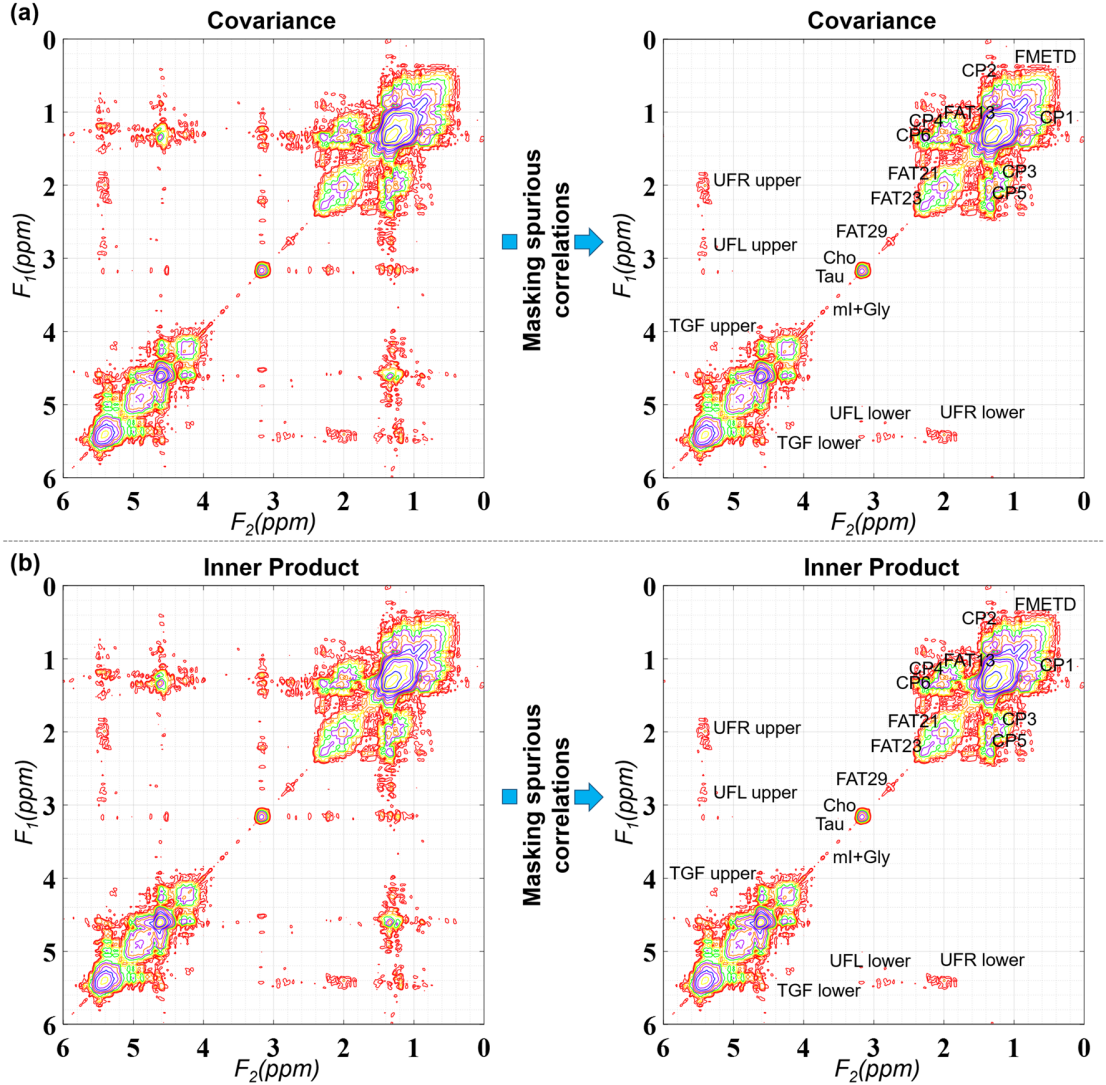
Masking spurious correlations in (**a**) CT and (**b**) IP based spectrum shown in this figure. Panels on left show contour plots with spurious correlations. Panels on the right show the spectrum after removing spurious correlations.

**Figure 4:**
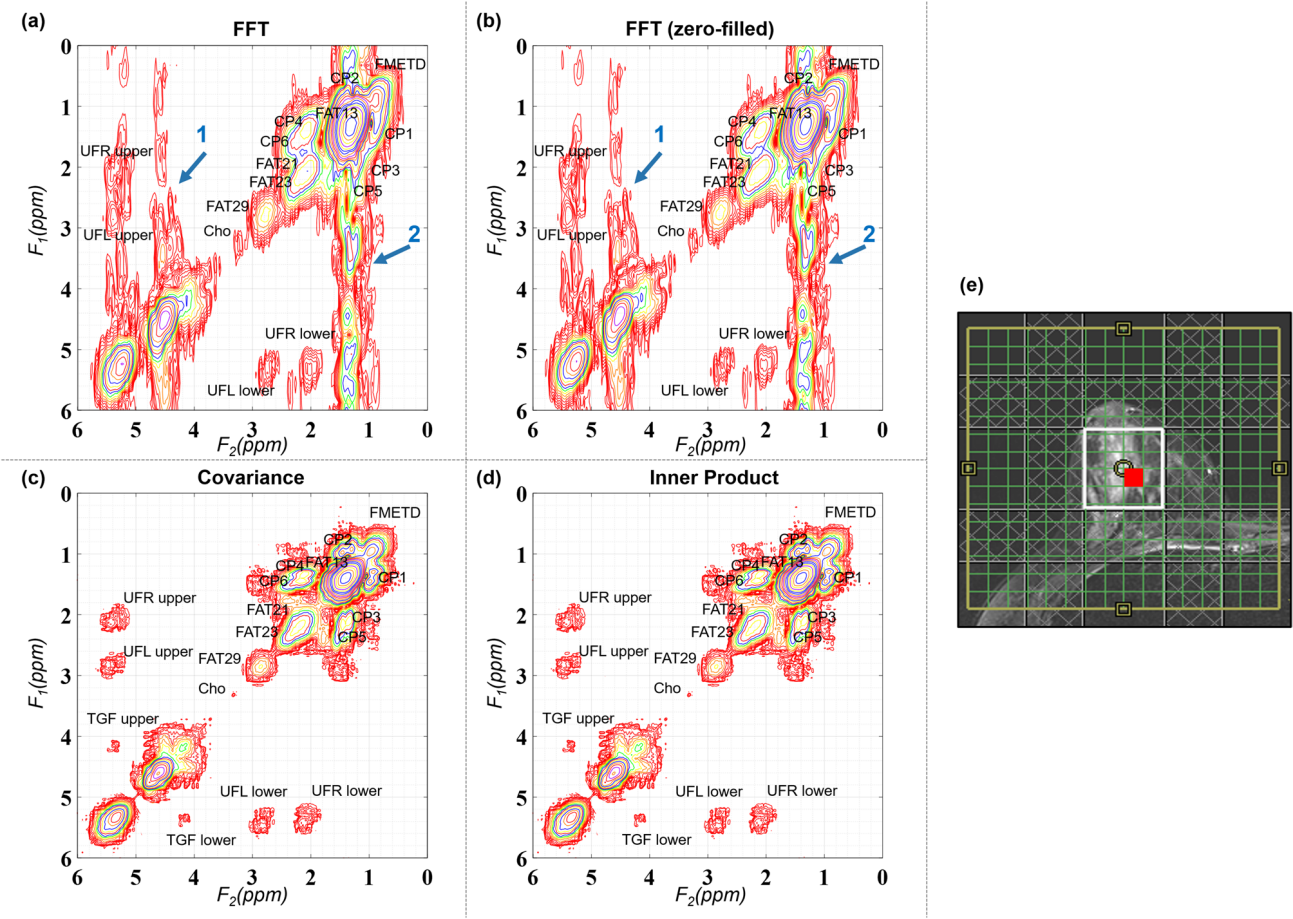
Reconstructed 2D COSY spectra from malignant lesions identified in 41-year-old patient (grade 3 invasive ductal carcinoma and ductal carcinoma in situ, estrogen receptor positive, progesterone receptor positive, her2 positive, ki-67 = 60% and BI-RADS 5): (**a**) FFT based spectrum, (**b**) FFT based spectrum after zero-filling along t_1_, (**c**) CT based spectrum, (**d**) IP based spectrum, (**e**) T_2_-weighted MR image with the white box representing the VOI placement. Red square shows the location of extracted spectrum. Arrows 1 and 2 show\ the effect of t_1_-ridging in (**a, b**).

Figure 3 shows the CT and IP 2D spectra of that shown in Fig. 2 after masking out the spurious correlations. Another 2D spectrum from malignant lesion identified in 41-year-old patient is shown in Fig. 4. Sections (a)–(d) shows the spectra generated using FFT, FFT with zero-filling along t_1_, CT and IP. T_2_-weighted MR image with the white box representing the VOI placement is shown in (e). Red square shows the location of extracted 2D spectrum. Arrows 1 and 2 show the effect of t_1_-ridging in (a) and (b).

### Choice of t_1_ points and acceleration feasibility

**Figure 5:**
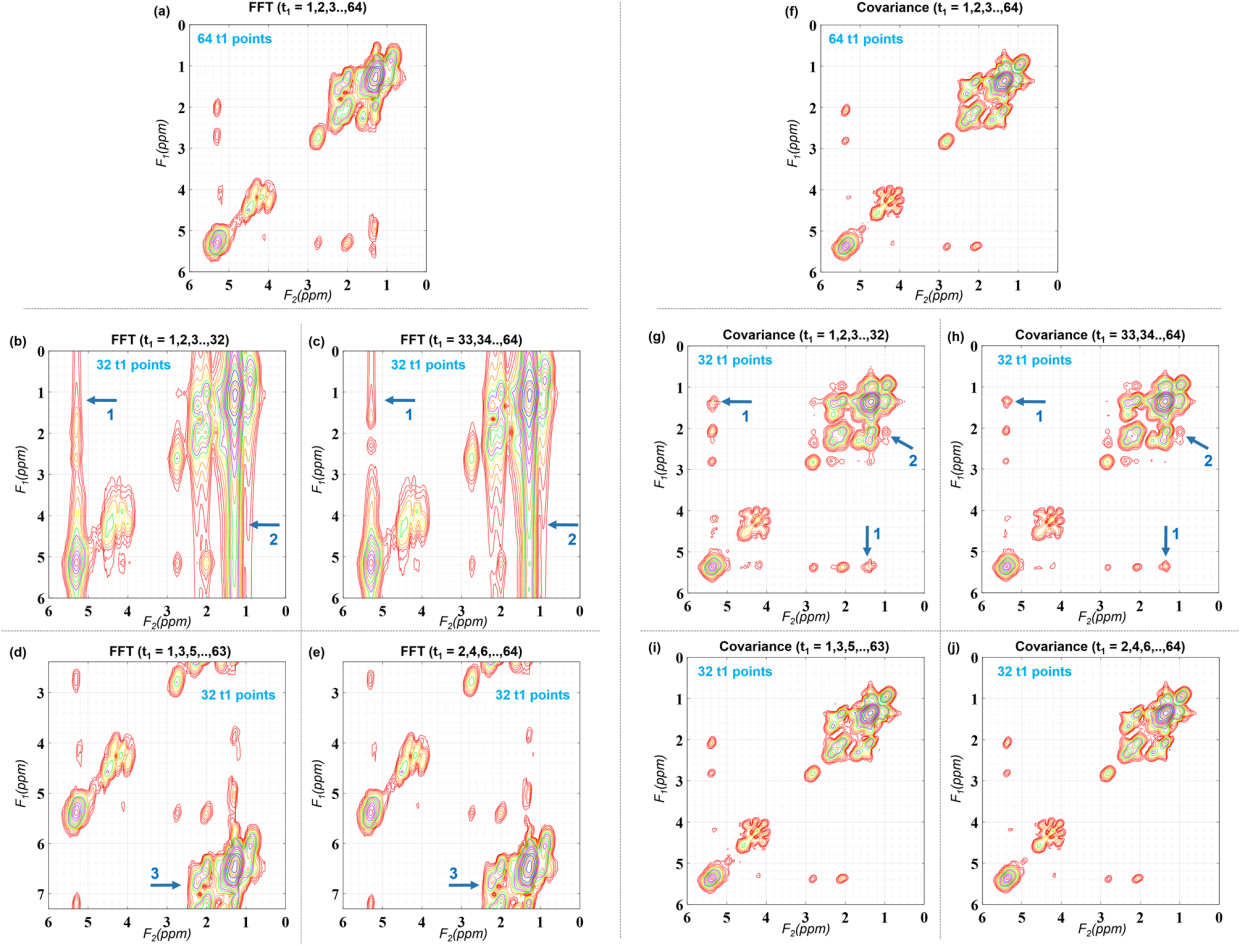
Effects of t_1_ sampling in FFT and CT based spectra. (**a**) FFT spectrum using 64 points along t_1_ ranging TEs from 35 to 85.4 ms at 800 μs intervals, giving 1250 Hz SW. (**b**) FFT spectrum using first 32 t_1_ points. (**c**) FFT spectrum using last 32 t_1_ points. Both (**b**) and (**c**) reduce the spectral resolution along t_1_ by half and show heavy t_1_-ridging effects (arrows 1 and 2). Sampling only (**d**) odd t_1_ points or (**e**) even t_1_ points for 2× acceleration halves the SW along t_1_ while retaining the same spectral resolution causing folding artifacts indicated by arrow 3. (**f**) CT spectrum using 64 points along t_1_ ranging TEs from 35 to 85.4 ms at 800 μs intervals, giving 1250 Hz SW. (**g**) CT spectrum using first 32 t_1_ points (t_1_ SW = 1250 Hz). (**h**) CT spectrum using last 32 t_1_ points (t_1_ SW = 1250 Hz). Arrows 1 and 2 in (**g**) and (**h**) points out the spurious correlations appearing in the spectrum. (**i**) Sampling every other t_1_ points starting from t_1_ = 1. Effective t_1_ SW = 1250 Hz. (**j**) Sampling every other t_1_ points starting from t_1_ = 2. Effective t_1_ SW = 1250 Hz.

Figure 5 shows the effect of different schemes of t_1_ sampling while using FFT and CT. The 2D spectrum here is the same spectrum as shown in Fig. 1, retrospectively undersampled along t_1_ to study feasibility of further acceleration. Figure 5a shows the conventional FFT spectrum using the full 64 points along t_1_ ranging TEs from 35 to 85.4 ms at 800 μs intervals, giving 1250 Hz SW. Choosing either first 32 or last 32 points as shown in (b) and (c) reduces the spectral resolution along F_1_ by half and shows heavy t_1_-ridging effects (arrows 1 and 2). Sampling every other t_1_ points for 2 × acceleration as shown in (d) and (e) on the other hand halves the SW along F_1_ while retaining the same spectral resolution causing folding artifacts indicated by arrow 3.

Panels (f)–(j) in Fig. 5 show the results of same cases as in panels (a)–(e) when CT is used instead of FFT. Arrows 1 and 2 in (g) and (h) points out the spurious correlations appearing in the spectrum. The results for IP are shown in Supplementary Figure 1 were the panels (a)–(e) correspond to the same cases as in panels (f)–(j) of Fig. 5.

### Quantitation comparison

**Figure 6:**
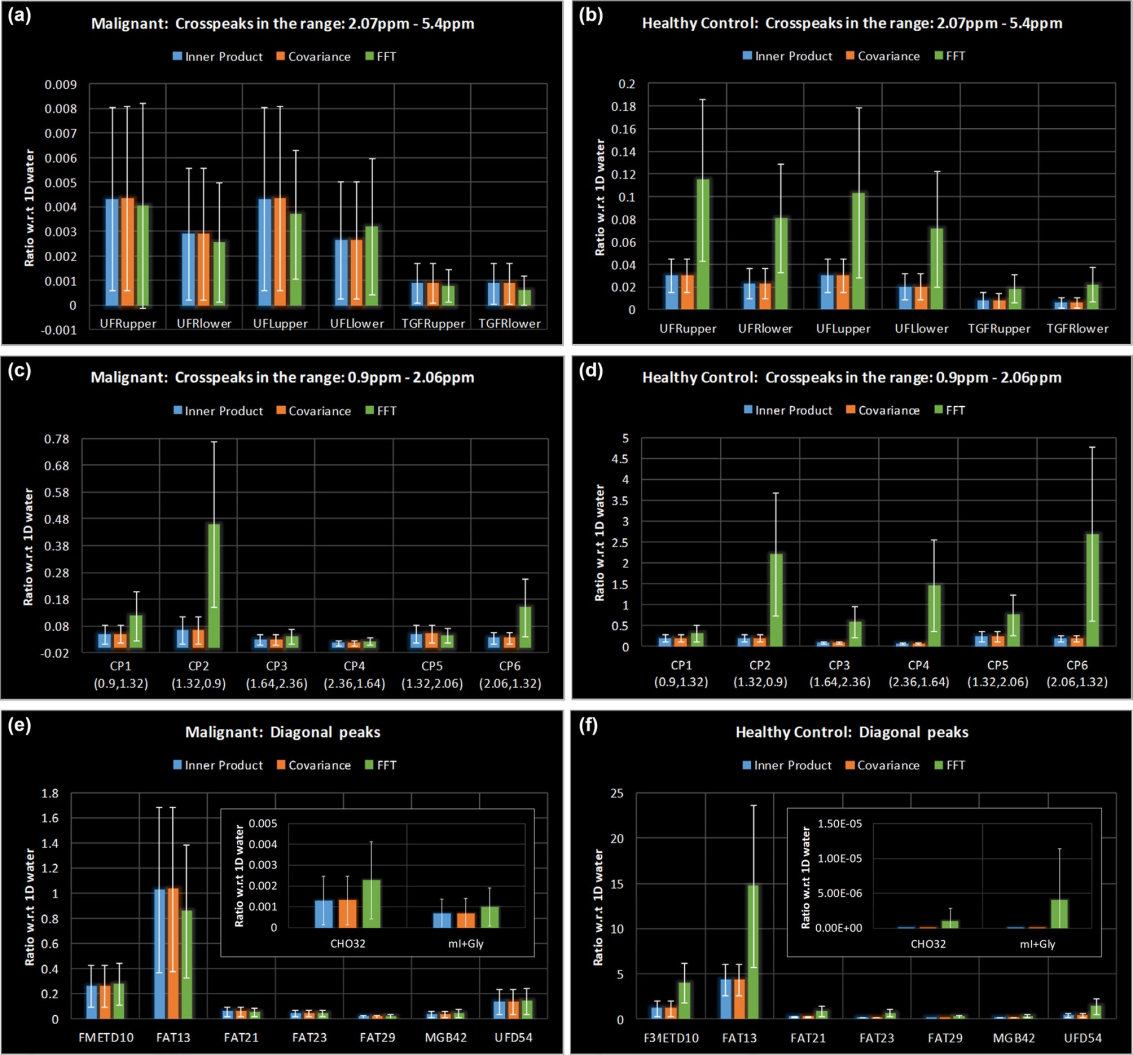
Mean (95% CI) of different metabolite and lipid ratios with respect to 1D water in both malignant (**a, c, e**) and healthy (**b, d, f**) breasts. Plots in (**a–d**) show the ratios for different cross peaks while (**e, f**) show the ratios for diagonal peaks in the spectrum.

Figure 6 shows bar graphs comparing the mean (95% CI) of different metabolite and lipid ratios with respect to 1D water peak area in both malignant ((a), (c) and (e)) and healthy ((b), (d) and (f)) breasts. Plots in (a) to (d) show the ratios for different cross peaks while (e) and (f) shows the ratios for diagonal peaks in the spectrum. A statistically significant difference between the estimation of ratios from IP, CT and FFT was determined by Brown-Forsythe ANOVA for CP2 (*F*(2,9.99) = 5.399, *p* = 0.026) in malignant group, and for CP2 (*F*(2,9.13) = 6.477, *p* = 0.018), CP3 (*F*(2,9.23) = 6.434, *p* = 0.018), CP4 (*F*(2,9.02) = 5.569, *p* = 0.027) and CP5 (*F*(2,9.05) = 5.012, *p* = 0.034) in healthy group. However, the Games-Howell post hoc test showed that the difference in the estimation of the ratios between any two methods among IP, CT and FFT were not statistically significant.

**Figure 7:**
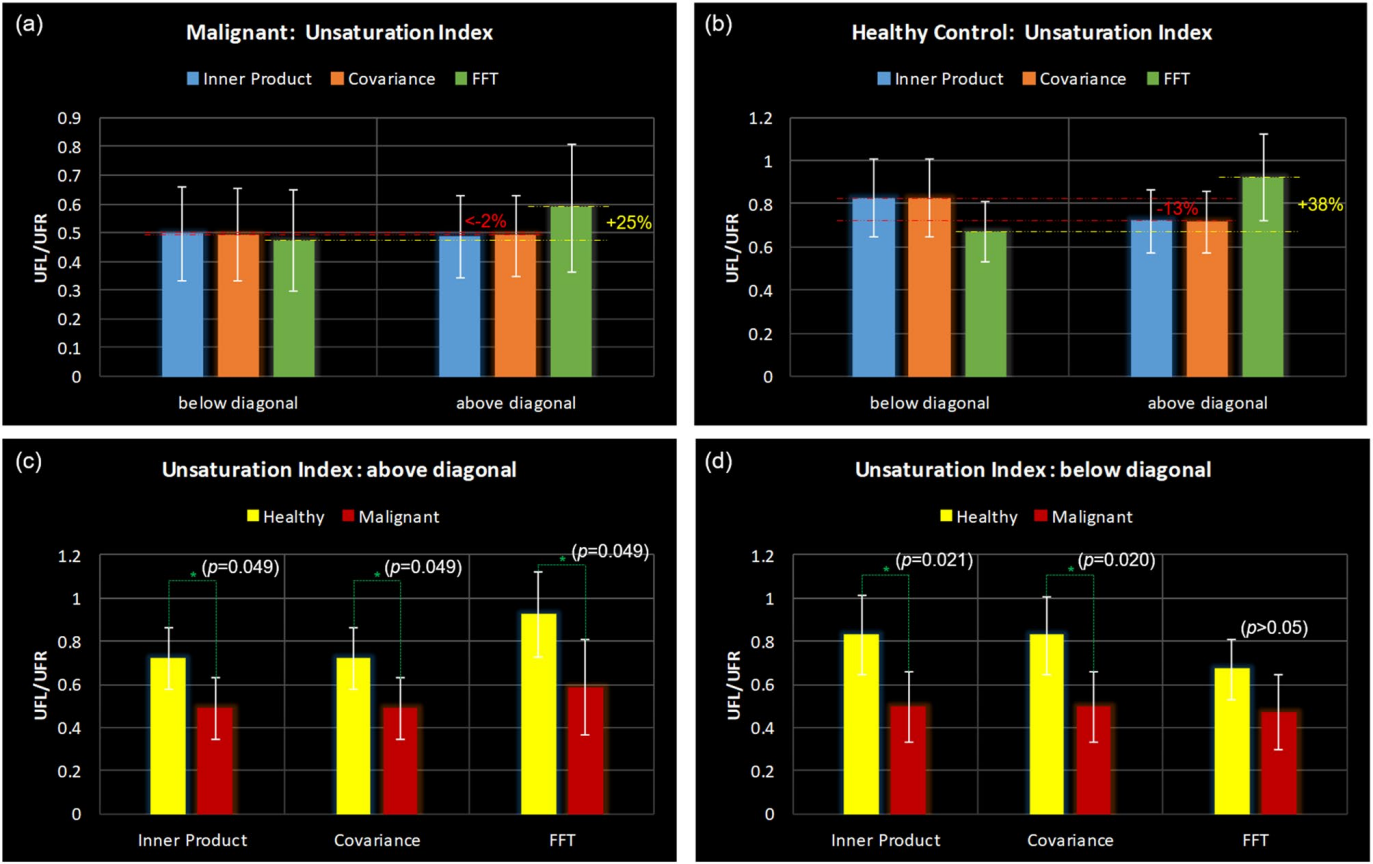
Bar graphs comparing the unsaturation index between IP, CT and FFT as well as across healthy and malignant groups. Comparison between IP, CT and FFT when the unsaturation index is computed from cross peaks above and below the diagonal in (**a**) malignant group, (**b**) healthy group. (**c**) Unsaturation index from cross peaks above the diagonal compared between healthy and malignant groups. (**d**) Unsaturation index from cross peaks below the diagonal compared between healthy and malignant groups.

Figure 7 shows bar graphs comparing the UI between IP, CT and FFT as well as across healthy and malignant groups. (a)–(b) show the results for malignant and healthy groups compared between IP, CT and FFT when the UI is computed from cross peaks above and below the diagonal. (c)–(d) show UI from cross peaks above and below the diagonal compared between healthy and malignant groups. Statistically significant differences (p < 0.05) were observed between healthy and malignant groups for UI computed from cross peaks both above and below diagonal for IP and CT, and above the diagonal for FFT. The measured values of UI from the cross peaks above and below the diagonal were very close for IP and CT with difference being < 2% for malignant and 13% for healthy, whereas they were much larger for FFT with a 25% difference for malignant and 38% difference for healthy as shown in (a) and (b).

## Discussion

Due to the scan time limitations, conventional 2D L-COSY spectrum is acquired with limited number of t_1_ points. Even with non-uniform sampling and CS based reconstruction, 64 t_1_ points are usually used to achieve reasonable scan time. This results in poor spectral resolution along F_1_. In this work, prospectively undersampled 5D EP-COSI data were reconstructed using GS-CS and 2D COSY spectra from multiple locations in malignant and healthy breast masses were analyzed using FFT, CT and IP for enhanced F_1_ spectral resolution, reduced t_1_-ridging, and acceleration feasibility for faster scan times.

### Limitations of FFT based spectrum

One the main requirements of FFT based analysis is that the time increments (∆t_1_) for t_1_ points should fulfill Nyquist theorem (∆t_1,Nyq_ = 1/SW) to avoid aliasing artifacts. Doubling the duration of t_1_ increment for example, results in halving the SW along F_1_ (625 Hz instead of 1250 Hz) while retaining the same spectral resolution (~ 19.53 Hz) if 32 t_1_ points are collected for 2 × acceleration. This results in aliasing artifacts along F_1_ due to the lack of sufficient SW (see Fig. 5d, e).

On the other hand, if SW is retained by keeping ∆t_1_ = 800 μs while reducing the number of t_1_ increments to 32, it results in poorer spectral resolution (~ 39.1 Hz) (see Fig. 5b, c). Common approach of zero-padding before FFT achieves interpolation to a larger matrix size, increasing the digital resolution, without actually increasing the spectral resolution. It can also introduce ringing in the spectrum in case of discontinuity in the time domain (see Fig. 1). The ringing can however be minimized by applying appropriate filters before zero-padding.

### Advantages of CT and IP

An advantage of CT and IP over traditional 2D FFT is that the indirect dimension of **S** is not required to be sampled with a time increment ∆t_1_ that fulfills the Nyquist theorem. Furthermore, if number of t_1_ point needs to be reduced to accelerate the acquisition, a wider range of t_1_ evolution times can be used with CT and IP without sacrificing the SW, since the SW along F_1_ in CT and IP will be equal to that of F_2_ dimension. It is also reported that probing wider range of t_1_ evolution times allows better discrimination between true and spurious spin correlations^31^. Unlike zero-filled FFT, CT and IP facilitate true spectral resolution enhancement along F_1_ as clearly shown in the results section. Even tough uniform increments along t_1_ are shown in results section to demonstrate acceleration feasibility of CT and IP in comparison with FFT, non-uniform sampling along t_1_ is also feasible with both IP and CT. The choice of specific set of increments needs further investigation and is the subject of future work. One approach would be to use the prior knowledge simulations to identify the set of t_1_ increments that will maximize the sensitivity of metabolites or lipids of low concentrations.

Another advantage of the CT and IP is the lack of t_1_-ridging artefacts. This results in cross peaks that are much better defined in the ppm range where t_1_-ridges are present in the FFT spectrum, for example, the lipid cross-peaks near 1.3 ppm along F_2_ (see Figs. 1, 2, 3, 4). Consequently, the quantitation of these cross-peaks improves substantially. It was observed that there is a larger difference between the ratios of cross peaks on either side of the diagonal in FFT as opposed to CT and IP. This resulted in a larger difference between these cross-peaks on either side of the diagonal with FFT as opposed to CT and IP, despite the symmetric property of the COSY spectra (see Figs. 6c, d, 7). This can cause variation in the measures of UI which is one of the important potential biomarkers available in the COSY spectra depending on whether upper cross peaks or lower cross peaks are used for its computation. Ideally, the ratios should be similar on either side of the diagonal, but the lower resolution along F_1_, and t_1_ ridging effects could influence these measures. Earlier studies have reported this measure from the cross peaks either above or below the diagonal or an average of the peaks on either side^16,32^. Statistically significant difference among IP, CT and FFT were observed in the cross peaks CP2, CP3, CP4 and CP5, especially in healthy controls. In the ratios from malignant tissues, only the difference in CP2 was statistically significant. This may be because of the fact that the healthy breast tissues generally have dominant fat peaks compared to malignant tissues. As a result, the chances of t_1_ ridging is higher near the aforementioned cross peaks in healthy tissues.

Furthermore, CT and IP based approaches give substantial gain in actual spectral resolution. The CT and IP based spectra gives the same bandwidth along F_1_ as that of F_2_. Therefore, these methods gave a spectral resolution of ~ 2.32 Hz along F_1_, while FFT based spectra had a spectral resolution of ~ 19.53 Hz in the experiments shown in results section. The metabolite peaks between 3 and 4 ppm were therefore much better resolved with these techniques compared to FFT in spectra from malignant tissues (see Figs. 2, 3, 4). This is especially important considering the ability of 5D EP-COSI in vivo detection and quantitation of metabolites like mI and Gly, in addition to measuring lipid-based biomarkers^16^. Reports from ex vivo breast cancer tissues have also shown the role of these metabolites including mI, Gly and Cho in identifying malignancy^5,33^. Even when not as resolved as that of CT and IP, the FFT based spectra also showed higher intensities in 3 ppm-4 ppm range in malignant tissues. Hence, the results of quantitation showed that these metabolites were elevated in malignant breast tissues compared to healthy ones with all three methods (see Fig. 6e, f). Since mI and Gly are separated by only 0.006 ppm, it requires < 1 Hz spectral resolution to disentangle them at 3 T. Despite this factor, it can be argued that the combined effect of high spectral resolution (~ 2.32 Hz) and absence of t_1_-ridging made the identification and analysis of these potential bio markers for breast cancer in CT and IP much less ambiguous as compared to FFT.

### Limitations of CT and IP

The main challenge with CT and IP is the possibility of spurious cross-peaks which has intensities above the noise level despite not representing the spin correlations. While this might arise from limited number of t_1_ points, we have observed that the FFT spectra with heavy t_1_-ridging also has tendency for stronger spurious correlations in CT and IP. Interestingly, the relationship between the number of t_1_ increments and spurious correlation appeared to be also dependent on the increment time used (see Fig. 5). However, the deterministic nature of the resonance positions and sampling pattern makes it easy to identify these false cross peaks^31^. Furthermore, since these spurious correlations generally appeared away from the true resonant positions, it was less problematic compared to effects of t_1_-ridging in FFT based spectra. It has also been shown that the false cross peaks can be easily identified by performing the correlation experiments with various mixing times since the artifacts should not be affected by the mixing time^34^. While two fold acceleration is demonstrated in the results, further acceleration introduced more artifacts in the form of spurious correlations. Even though the CT and IP based spectra should be reliable to measure known correlations, assessment of new cross-peaks should therefore need careful analysis to discard the possibility of spurious correlations.

### Difference in CT and IP

While both CT and IP based spectra appeared almost identical, a notable difference was near residual water at 4.7 ppm. This is as expected since the theory of inner-product based transformation is designed to be robust against the choice of central frequency. Since our spectra were all centered at water, the frequencies near water undergo limited oscillation with increasing the evolution time which weakens the vanishing mean assumption in CT. IP on the other hand doesn’t have this requirement and hence argued to be more robust to the influence of the central frequency. However, in our application, the residual water was not important due to the water suppressed acquisition. Though the level of water itself in ratio with fat has been reported to be of importance in malignant breast cancers, this is usually calculated form the associated non-water suppressed 1D acquisition for eddy-current correction and coil combination. Therefore, both CT and IP served the purpose in a very identical manner throughout our experiments. However, it may be noted that the choice between the two should also be based on the choice of central frequency in the spectrum.

## Conclusion

Reconstruction of in vivo 5D EP-COSI using CT and IP is presented in this work showing enhanced F_1_-resolution of COSY in comparison with FFT based spectral analysis. With CT and IP, we were able to achieve ~ 2.32 Hz spectral resolution along F_1_ dimension as compared to ~ 19.53 Hz resolution in FFT based spectrum. The effect of t_1_-ridging artifacts commonly seen in FFT spectra was not observed in CT or IP. Consequently, both CT and IP showed well defined and symmetrical cross-peaks on either side of the diagonal as evident by the quantified ratios of the lipid cross-peaks above and below the diagonal. Furthermore, CT and IP were found to permit wider range of t_1_ increments without affecting the actual SW along F_1_. This is particularly advantageous in spectral analysis of metabolite and lipid biomarkers including unsaturation index for breast cancer while making significant gains in scan time.

### Supplementary Information

Supplementary Information.

## Data Availability

The datasets generated and/or analyzed during the current study are not publicly available due to ethical and data protection restrictions, but are available from corresponding author on reasonable request and subject to an institutional data sharing agreement.
