# Supplementary material for: Enhanced spectral resolution for correlated spectroscopic imaging using inner-product and covariance transform: a pilot analysis of metabolites and lipids in breast cancer in vivo
Source: Sci Rep. 2023 Oct 5;13:16809. doi: 10.1038/s41598-023-43356-8 (PMC10556085; doi:10.1038/s41598-023-43356-8)
Supplement: Supplementary file 1 — Supplementary Information. [file 41598_2023_43356_MOESM1_ESM.docx]

## SUPPORTING INFORMATION

**Figure S1**. Effects of t_1_ sampling in IP based spectrum. (a) IP spectrum using 64 points along t_1_ ranging TEs from 35ms to 85.4ms at 800μs intervals, giving 1250Hz SW. (b) IP spectrum using first 32 t_1_ points (t_1_ SW = 1250Hz). (c) CT spectrum using last 32 t_1_ points (t_1_ SW = 1250Hz). Arrows 1 and 2 in (b) and (c) points out the spurious correlations appearing in the spectrum. (d) Sampling every other t_1_ points starting from t_1_=1. Effective t_1_ SW = 1250Hz. (e) Sampling every other t_1_ points starting from t_1_=2. Effective t_1_ SW = 1250Hz. Figures in all panels are displayed in the same scale.
